# Application of ^68^Ga‐ and ^177^Lu‐Labeled FAP Inhibitor in Evaluation and Treatment of Cardiac Fibrosis After Myocardial Infarction

**DOI:** 10.1002/mco2.70198

**Published:** 2025-05-16

**Authors:** Yiheng Zhao, Xiangyu Su, Boyang Xiang, Shuchen Zhang, Xiang Zhou

**Affiliations:** ^1^ Department of Cardiology The Second Affiliated Hospital of Nanjing Medical University Nanjing China; ^2^ Department of Cardiology The Second Affiliated Hospital of Soochow University Suzhou China

**Keywords:** cardiac fibrosis, ^68^Ga‐FAPI, ^177^Lu‐FAPI, myocardial infarction

## Abstract

^68^Ga and ^177^Lu‐labeled fibroblast activation protein inhibitor (FAPI) have been introduced for the diagnosis and treatment of multiple malignant and non‐malignant diseases. While several studies have examined the application of ^68^Ga‐FAPI in myocardial infarction (MI), research on the use of ^177^Lu‐FAPI for the treatment of MI is still scarce. In this study, we evaluated the effects of ^68^Ga‐FAPI and ^177^Lu‐FAPI in cardiac fibrosis after MI using permanent coronary artery ligation rat models. ^68^Ga‐FAPI‐04 effectively targeted fibroblasts within the MI area. Rats treated with ^177^Lu‐FAPI‐04 had a significant increase in left ventricular ejection fraction at 28 days post‐MI, with no obvious kidney or liver toxicity. Magnetic resonance imaging and histological analysis revealed a reduced fibrotic area in the ^177^Lu‐FAPI group. ^177^Lu‐FAPI‐04 exerted its therapeutic effect by suppressing activation and inducing apoptosis of fibroblasts. In summary, we demonstrated that ^177^Lu‐FAPI‐04 could effectively target FAP and eliminate activated fibroblasts after MI, thereby contributing to the development of new strategies for the treatment of MI.

## Graphical abstract

Our study evaluates the specific accumulation of ^68^Ga‐FAPI‐04 in infarcted hearts and suggests that treatment with ^177^Lu‐FAPI‐04 may enhance cardiac function by modulating fibroblast activation and apoptosis, thereby reducing cardiac fibrosis after myocardial infarction.

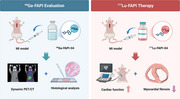

## 1 Introduction

Myocardial infarction (MI) is a severe cardiovascular disease with high mortality [1]. Ventricular remodeling after MI is a key factor leading to the development of heart failure (HF), which has an important influence on patient prognosis. Myocardial cell death as a result of MI activates innate immune pathways and the release of cytokines, chemokines, and adhesion molecules, which triggers a series of inflammatory reactions [2].

Cardiac fibroblasts are activated in response to ischemic injury following MI and play an important role in extracellular matrix (ECM) deposition and myocardial fibrosis [3]. Upregulation of fibrotic cytokines, such as fibroblast growth factors and transforming growth factors, results in the differentiation of resident cardiac fibroblasts and other interstitial cells into myofibroblasts, which express multiple proteins including α‐smooth muscle actin (α‐SMA), vimentin, and periostin and create scars by secreting ECM [4, 5]. Myofibroblasts initiate the reparative wound healing response after exposure to pathological stimuli. The continuous activation of abnormal myofibroblasts mediated by various fibrogenic mediators is a critical event associated with the development of fibrosis [6]. Although reparative scar formation prevents myocardial rupture and maintains regular myocardial architecture at an early stage, myofibroblast overactivation can cause excessive ECM secretion, distort the cardiac structure, and promote cardiac dysfunction, thus contributing to the progression of MI to HF [7, 8].

Fibroblast activation protein (FAP) is a transmembrane serine protease expressed in fibroblasts [9]. The protease is not generally expressed in normal adults but is expressed in multiple pathological processes such as inflammation, tissue remodeling, and fibrosis [10, 11]. In particular, FAP is expressed in activated but not in resting fibroblasts [12]. Because of its low expression in normal adult tissues, FAP has been recognized as a novel molecular diagnostic and therapeutic target for diseases associated with ECM remodeling.

Diverse quinoline‐based FAP inhibitor (FAPI) tracers have recently been developed for positron emission tomography (PET)/computed tomography (CT) imaging, and radiolabeled FAPI tracers have been used in PET imaging for tumor diagnosis [13]. Multiple studies have also suggested that there is high uptake of FAPI in MI [14], chemotherapy‐induced cardiotoxicity [15], immune checkpoint inhibitor‐associated myocarditis [16], and other cardiovascular diseases [17]. In addition, recent research indicated that FAPI could be used in FAP‐targeted radioligand therapy in malignant diseases [18]. However, the therapeutic application of FAPI in cardiovascular diseases is still scarce, and drugs that can promote cardiac repair in the clinic are still lacking. Based on FAPI, this study aimed to validate the feasibility of ^68^Ga‐labeled FAPI for imaging activated fibroblasts after MI and evaluate the potential of ^177^Lu‐labeled FAPI for the treatment of MI.

## 2 Results

### 2.1 Radiopharmaceutical Synthesis

Based on high‐performance liquid chromatography (HPLC) analysis of the crude product, the radiochemical yields of ^68^Ga‐FAPI‐04 and ^177^Lu‐FAPI‐04 were 49% and 70%, respectively. The radiochemical purity of ^68^Ga‐FAPI‐04 was ≥98% after purification and ^177^Lu‐FAPI‐04 was consistently >98% without purification. The activities of ^68^Ga‐FAPI‐04 and ^177^Lu‐FAPI‐04 was 6.81 and 12.82 Mbq/nmol, respectively.

### 2.2 Specific Accumulation of ^68^Ga‐FAPI in Infarcted Hearts

**FIGURE 1:**
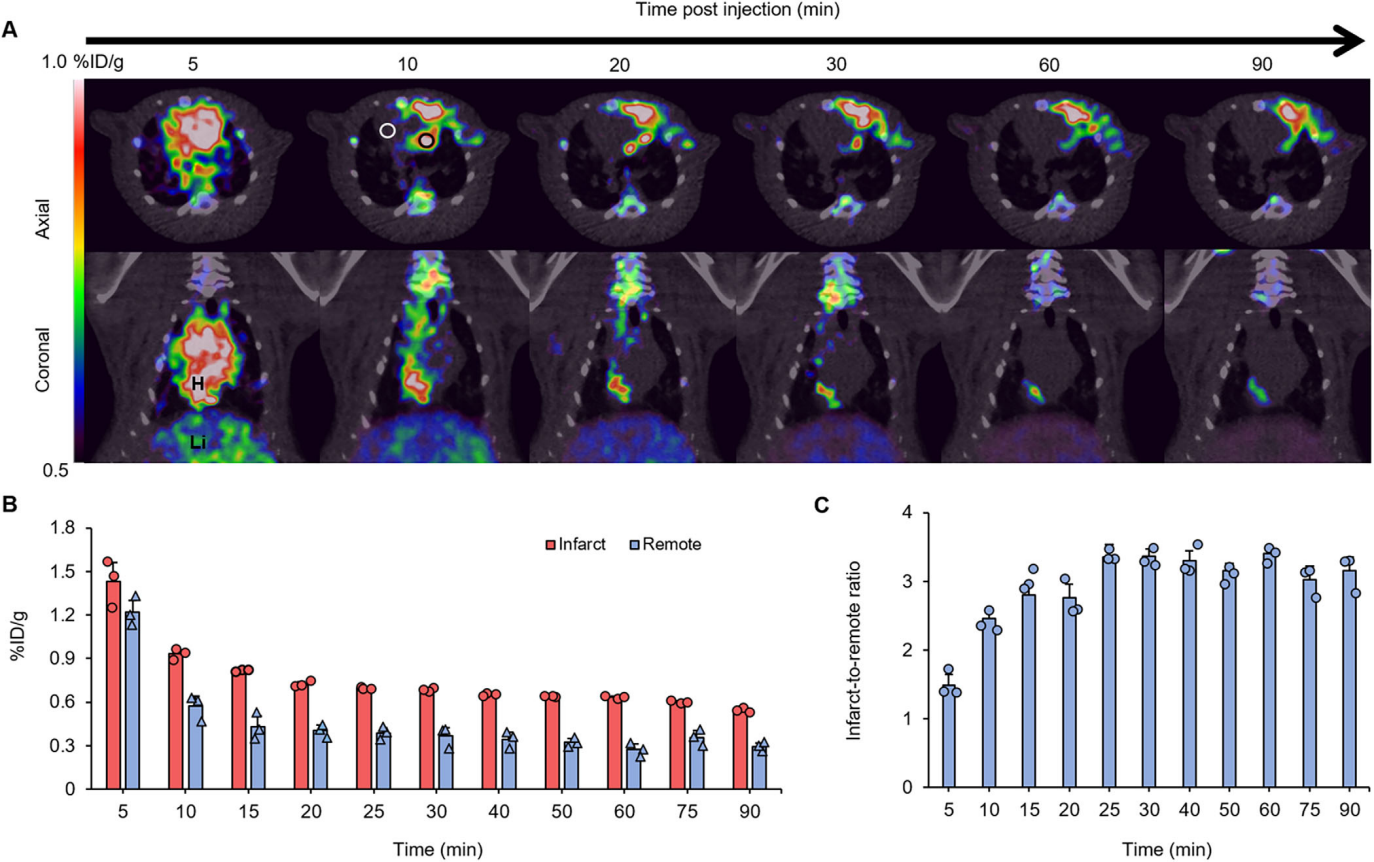
Dynamic ^68^Ga‐FAPI‐04 PET/CT scanning. (A) Dynamic PET/CT images of rats at 7 days after MI (axial and coronal sections). Black and white circles at 10 min after tracer injection indicate regions of interest of infarcts and remote myocardium, respectively. H: heart; Li: liver. (B) ^68^Ga‐FAPI‐04 uptake in infarcted and remote non‐infarcted myocardium overtime (*n* = 3). (C) PET image‐based infarct‐to‐remote tracer uptake ratio over time (*n* = 3).

Cardiac PET/CT imaging was performed to further explore the in vivo characteristics of ^68^Ga‐FAPI‐04. Dynamic PET/CT images from 5–90 min after tracer injection are shown in Figure 1A. ^68^Ga‐FAPI‐04 uptake peaked within a short time and then steadily decreased (Figure 1B). ^68^Ga‐FAPI‐04 was cleared steadily from infarcted areas and more quickly from distant normal tissues, leading to increasing infarct‐to‐remote ratios over time (Figure 1C).

**FIGURE 2:**
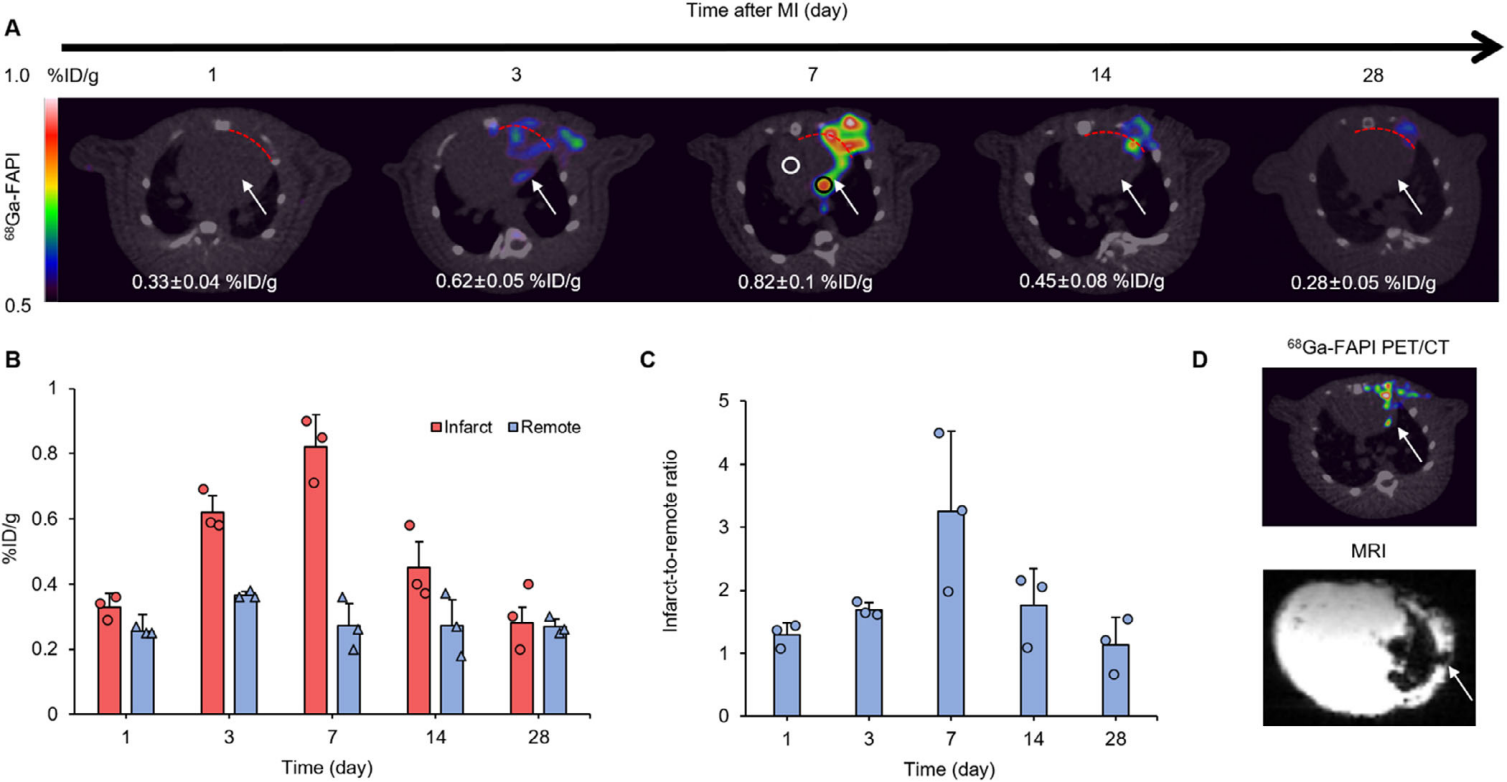
^68^Ga‐FAPI‐04 PET/CT imaging at different time points. (A) Static axial PET/CT images of the same MI rat at 1, 3, 7, 14, and 28 days after coronary ligation. Black and white circles at 7 days after coronary ligation indicate regions of interest of infarcted and remote myocardium, respectively. Dashed lines represent demarcation between surgical wound and heart. (B) Tracer uptake in infarcted and remote myocardium at different time points (*n* = 3). (C) PET image‐based infarct‐to‐remote tracer uptake ratio over time (*n* = 3). (D) In vivo ^68^Ga‐FAPI‐04 PET/CT and corresponding ex vivo MRI images in another MI rat. Infarcted area illustrated in MRI image was consistent with the region of tracer accumulation in PET/CT.

Figure 2A shows PET/CT images from a representative rat in the MI group at different time points. ^68^Ga‐FAPI‐04 uptake was observed at the infarct region, peaking (0.82 ± 0.1 %ID/g) 7 days after surgery, and then falling to background levels (0.28 ± 0.05 %ID/g) around 4 weeks after coronary ligation (Figure 2B). The highest infarct‐to‐remote ratio occurred 7 days after coronary ligation (3.25 ± 1.27, Figure 2C). Moreover, ex vivo 7 T magnetic resonance imaging (MRI) examination of another MI heart demonstrated that the region with ^68^Ga‐FAPI‐04 accumulation was consistent with the infarct region revealed by MRI scanning (Figure 2D).

**FIGURE 3:**
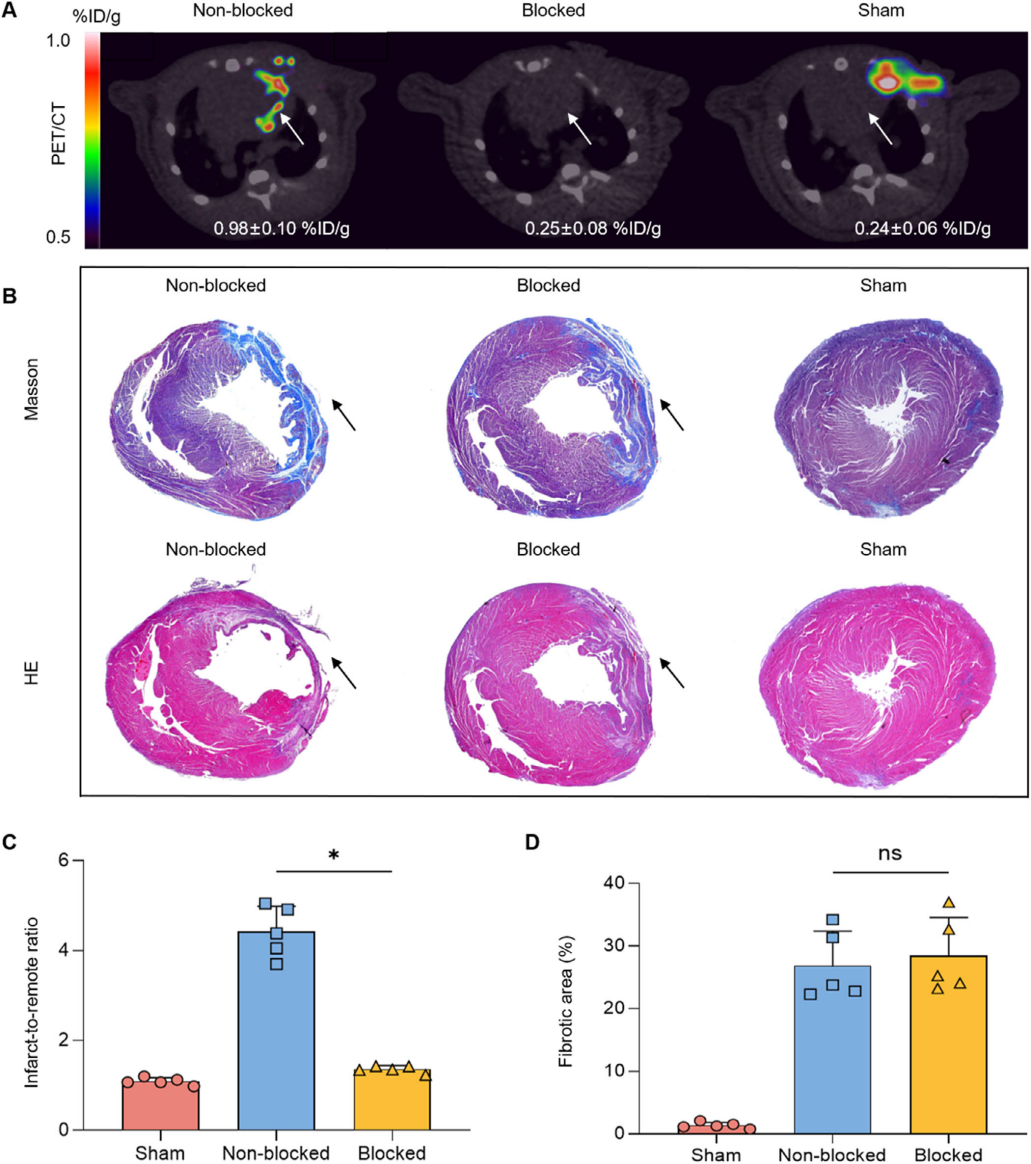
Blocking experiments: (A) Axial PET/CT images from non‐blocked, blocked, and sham‐operated rats at 7 days after surgical operation. (B) Masson's and HE staining of parallel sections. Infarct areas in pathological sections are indicated by black arrows. (C) PET image‐based infarct‐to‐remote tracer uptake ratio (*n* = 5, *p < 0.05). (D) Quantitative analysis of fibrotic area based on Masson's staining (*n* = 5, ns = no significant difference).

Blocked and non‐blocked rats were compared at 7 days post‐MI (Figure 3A). Figure 3B shows Masson's and hematoxylin–eosin (HE) staining of parallel sections. The infarcted myocardium had a relatively high infarct‐to‐remote tracer uptake ratio at 7 days after coronary ligation (4.55 ± 0.6), but the uptake of ^68^Ga‐FAPI‐04 was dramatically reduced after co‐injection of non‐labeled FAPI‐04 (0.25 ± 0.08 %ID/g) (*p* < 0.001, Figure 3C). In other words, the saturation of FAP by FAPI‐04 decreased tracer uptake in the surgical wound to background levels. Fibrotic proportions in the non‐blocked, blocked, and sham groups are shown in Figure 3D. These results collectively suggested that FAPI‐04 could specifically bind to FAP and accumulate in infarcted myocardium.

### 2.3 ^177^Lu‐FAPI Therapy Improved Cardiac Function in MI Model

**FIGURE 4:**
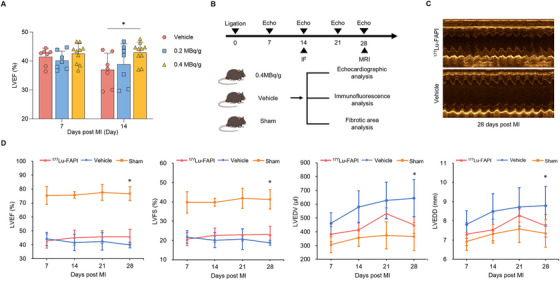
Echocardiographic analysis. (A) Measurement of LVEF in vehicle controls (*n* = 7) and MI rats injected with ^177^Lu‐FAPI‐04 (*n* = 7 for 0.2 MBq/g group and *n* = 10 for 0.4 MBq/g group). LVEF in rats in the 0.4 MBq/g group exhibited an increasing trend 14 days post‐MI. (B) Timeline of following experiment for ^177^Lu‐FAPI, vehicle, and sham groups. (C) Representative echocardiography images of rats in the ^177^Lu‐FAPI group and vehicle group at 28 days after coronary ligation. (D) Echocardiographic analyses of LVEF, LVFS, LVEDV, and LVEDD at 7, 14, 21, and 28 days after MI (*n* = 11 for ^177^Lu‐FAPI group, *n* = 9 for vehicle group, and *n* = 9 for sham group). ^177^Lu‐FAPI‐04 was injected at 7 days post‐MI. There were no significant differences in cardiac function parameters among the three groups before coronary ligation, and no statistically significant difference in cardiac function parameters between the ^177^Lu‐FAPI and vehicle groups before ^177^Lu‐FAPI‐04 treatment. IF, Immunofluorescence; MRI, magnetic resonance imaging; LVEF, left ventricular ejection fraction; LVFS, left ventricular fractional shortening; LVEDV, left ventricular end‐diastolic volume; LVEDD, left ventricular end diastolic diameter (*p < 0.05).

Echocardiography was used to detect the therapeutic effect of ^177^Lu‐FAPI‐04 in MI rats. The vehicle‐treated controls and rats in 0.2 MBq/g group showed lower left ventricular ejection fraction (LVEF) 14 days after ligation (Figure 4A). In contrast, LVEF values of rats in the 0.4 MBq/g group exhibited an increasing trend at 14 days post‐MI (36.99 ± 5.73% vs. 43.05 ± 3.54%, *p* = 0.016). We thus speculated that a dose of 0.4 Mbq/g is effective and used this dose in subsequent experiments. Normal rats with comparable cardiac function parameters underwent coronary ligation and were allocated into ^177^Lu‐FAPI, vehicle, and sham groups. The subjects were injected with ^177^Lu‐FAPI‐04 (0.4 MBq/g) or normal saline, and their cardiac function was monitored on a weekly basis after injection. The experimental timeline and echocardiography are illustrated in Figure 4B,C. At 28 days after MI, the LVEF of MI rats was significantly lower than that in the sham group. Compared with vehicle controls, rats treated with ^177^Lu‐FAPI‐04 showed significant increases in LVEF (45.74 ± 5.37% vs. 39.83 ± 2.00%, *p* = 0.006) and left ventricular fractional shortening (LVFS) (23.00 ± 4.33% vs. 18.77 ± 1.28%, *p* = 0.011), while left ventricular end‐diastolic volume (LVEDV) (448.86 ± 61.03 µL vs. 642.78 ± 134.84 µL, *p* < 0.001). and left ventricular end diastolic diameter (LVEDD) (7.75 ± 0.58 mm vs. 8.79 ± 0.99 mm, *p* = 0.009) were markedly decreased (Figure 4D). The above findings revealed the positive impact of ^177^Lu‐FAPI‐04 therapy on cardiac function improvement.

### 2.4 ^177^Lu‐FAPI Therapy Reduced Myocardial Fibrosis

**FIGURE 5:**
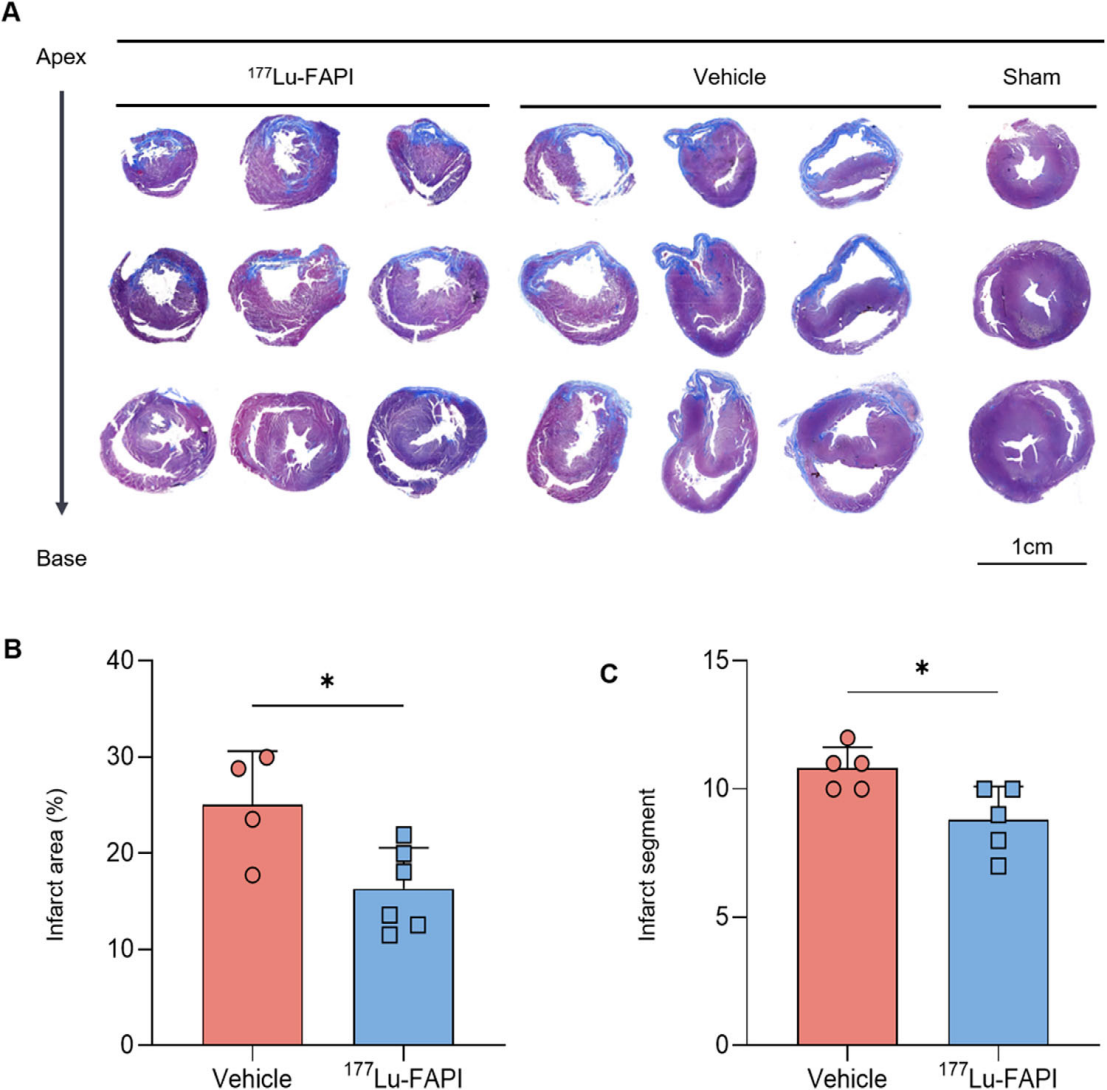
Assessment of the fibrotic area. (A) Representative Masson's staining of serial heart sections from apex to base at 28 days post‐MI. (B) Quantification of fibrotic area at 28 days after MI (*n* = 4 for vehicle group and *n* = 6 for ^177^Lu‐FAPI group). (C) Enumeration of infarct segments in MRI images (*n* = 5) (*p < 0.05).

Fibrotic proportions were evaluated in both ^177^Lu‐FAPI group and vehicle group considering its association with adverse ventricular remodeling and cardiac dysfunction. Rats were sacrificed 28 days after MI and three parallel sections were selected from the base to the apex of the hearts. Heart sections were examined by Masson's staining (Figure 5A). The fibrotic area in the ^177^Lu‐FAPI group (16.27 ± 4.3%) was significantly reduced compared with the vehicle group (25.02 ± 5.6%) (p < 0.023, Figure 5B). MRI examination revealed myocardial fibrosis in nine of 18 segments in the ^177^Lu‐FAPI group, which was significantly less than 11 of 18 segments in the vehicle group (*p* = 0.020, Figure 5C). All the above results supported that ^177^Lu‐FAPI therapy attenuated cardiac fibrosis after MI.

### 2.5 ^177^Lu‐FAPI Therapy Alleviates Myocardial Fibrosis by Regulating Fibroblast Activation and Apoptosis

**FIGURE 6:**
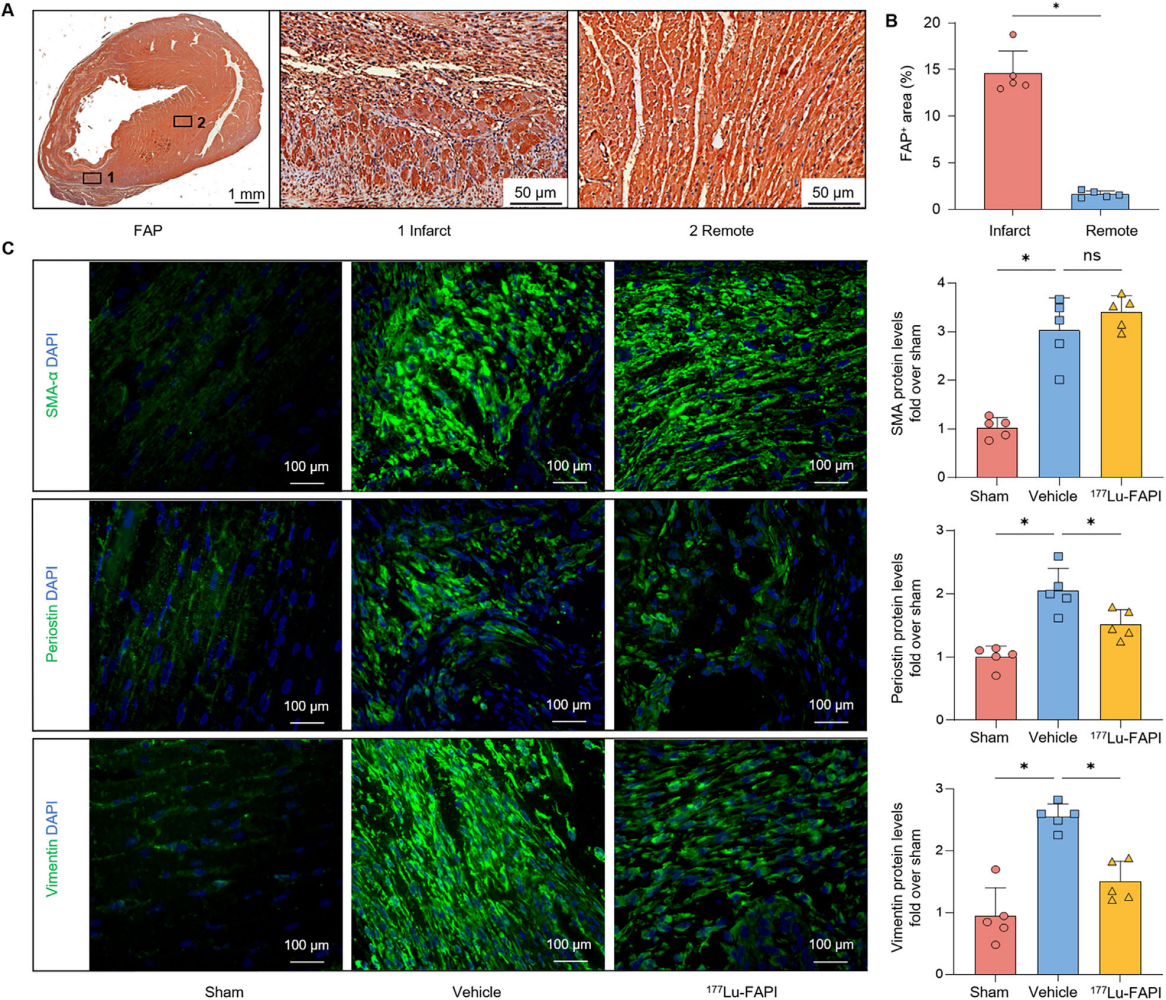
Pathological analysis of cardiac tissue. (A) Representative IHC images of FAP at 7 days post‐MI. Insets 1 and 2 show higher magnifications of infarct and remote normal myocardium, respectively. (B) Quantification of FAP^+^ cell area in infarct zone and remote normal myocardium at 7 days post‐MI (*n* = 5). (C) IF staining of α‐SMA, periostin, and vimentin in ^177^Lu‐FAPI, vehicle, and sham groups at 7 days after ^177^Lu‐FAPI‐04 injection (14 days post‐MI). Protein levels of α‐SMA, periostin, and vimentin levels were quantified and presented as fold change compared with sham‐operated rats. Sham group: Sham‐operated rats treated with normal saline (*n* = 5). Vehicle group: MI rats treated with normal saline (*n* = 5). ^177^Lu‐FAPI group: MI rats treated with ^177^Lu‐FAPI‐04 (*n* = 5). (ns = no significant difference, *p < 0.05).

Myocardial fibrosis was further assessed to investigate the mechanism by which ^177^Lu‐FAPI enhances cardiac function following MI. Isolated hearts from rats in the vehicle group were examined by immunohistochemical (IHC) staining at 7 days after MI. FAP^+^ cells were mainly clustered in the infarcted sectors, with almost no FAP^+^ cells in the distant normal myocardium (Figure 6A,B). Rats in the ^177^Lu‐FAPI, vehicle, and sham groups were sacrificed 14 days after MI (7 days after drug injection). Immunofluorescence (IF) imaging across all groups demonstrated a significant upregulation of activated fibroblast markers α‐SMA, periostin, and vimentin in MI rats (Figure 6C). In contrast, these markers were not prominently expressed in the normal myocardium. Although no apparent difference in the expression of α‐SMA was observed between the ^177^Lu‐FAPI group and vehicle group, periostin and vimentin exhibited a significant decrease after ^177^Lu‐FAPI injection. These observations revealed that ^177^Lu‐FAPI‐04 exerted its therapeutic effect by influencing fibroblast activation.

**FIGURE 7:**
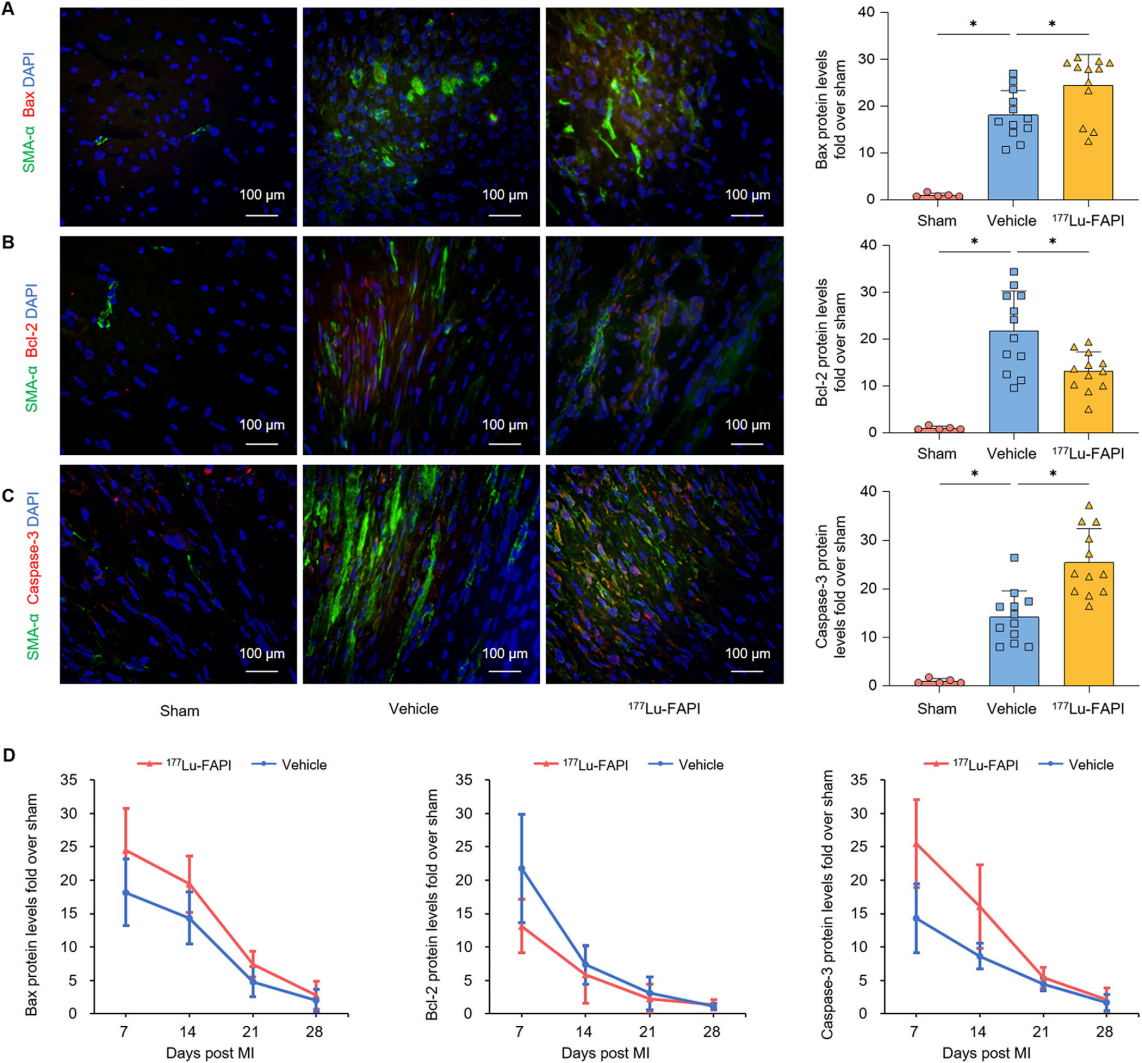
Detection of apoptotic proteins. (A–C) IF images of caspase‐3, Bax, and Bcl‐2 in different groups at 7 days post‐MI and corresponding quantitative analysis. (D) Apoptotic protein expression in ^177^Lu‐FAPI and vehicle groups over time. Sham group: Sham‐operated rats treated with normal saline (*n* = 5). Vehicle group: MI rats treated with normal saline (*n* = 12). ^177^Lu‐FAPI group: MI rats treated with ^177^Lu‐FAPI‐04 (*n* = 12) (*p < 0.05).

To evaluate the mechanism of ^177^Lu‐FAPI on fibroblasts, we collected heart sections from ^177^Lu‐FAPI, vehicle, and sham groups at different time‐points. The sections were analyzed using IF imaging to quantify the levels of apoptotic proteins in cells expressing α‐SMA. MI was found to increase the protein expression of Bax, Bcl‐2, and caspase‐3 (Figure 7A–C). The expression levels of Bax and caspase‐3 were significantly elevated in the ^177^Lu‐FAPI group compared to the MI group. Moreover, rats treated with ^177^Lu‐FAPI‐04 had lower expression of Bcl‐2. All these marker proteins decreased overtime and reduced to background levels at 28 days after MI (Figure 7D). Taken together, these results indicated ^177^Lu‐FAPI‐04 can attenuate cardiac fibrosis through the induction of fibroblast apoptosis.

### 2.6 Biodistribution and Toxicity

**FIGURE 8:**
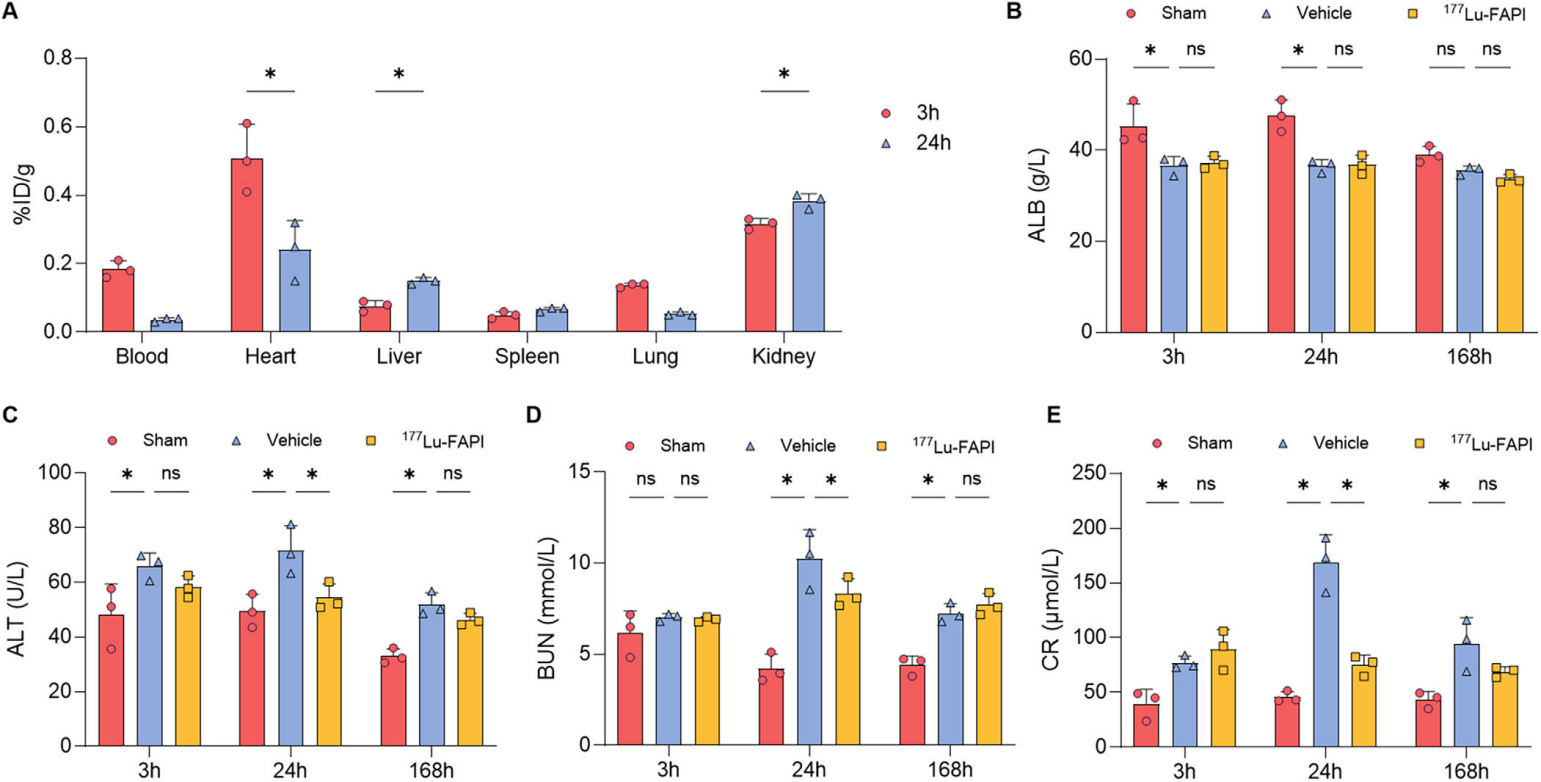
Radiopharmaceutical uptake and biochemical analysis. (A) ^177^Lu‐FAPI‐04 uptake at 3 and 24 h post‐drug injection (*n* = 3). (B–E) Biochemical analysis of ^177^Lu‐FAPI, vehicle, and sham groups (*n* = 3). ALB, albumin; ALT, alanine aminotransferase; BUN, blood urea nitrogen; CR, creatinine.

The biodistribution and toxicity of ^177^Lu‐FAPI‐04 were also examined in this study. The biodistribution of ^177^Lu‐FAPI‐04 is shown in Figure 8A. Relatively high ^177^Lu‐FAPI accumulation was seen in the heart at 3 h post‐injection of ^177^Lu‐FAPI‐04, and this decreased significantly at 24 h post‐administration. In contrast, the accumulation of ^177^Lu‐FAPI‐04 in the liver and kidney increased 24 h after injection.

Biochemical analysis revealed notable variations in ALT, BUN, and CR levels between the vehicle and sham groups 168 h post‐injection. However, no significant alteration was observed in ALB levels. CR levels exhibited a transient increase in the vehicle group at 24 h after injection. Nevertheless, there was no significant difference in blood biochemical parameters between the 177Lu‐FAPI and vehicle groups at 168 h post‐injection (Figure 8B–E). None of the rats died during the experiments. These data suggested that ^177^Lu‐FAPI‐04 might serve as a safe agent in the treatment of MI.

## 3 Discussion

Quiescent myocardial fibroblasts continue to secrete ECM to maintain regular myocardial function; however, cardiomyocyte death induced by MI triggers a series of inflammatory responses and activates fibroblasts [2]. Activated fibroblasts express FAP following MI, which plays an important role in cardiac repair and remodeling [19]. In the current study, we evaluated FAP expression in MI rats using PET/CT, which showed obvious ^68^Ga‐FAPI‐04 uptake in the infarcted myocardium, while the remote normal myocardium showed only transient tracer accumulation. Quantitative analysis showed the highest infarct‐to‐remote tracer ratio at 1 h post‐tracer injection. MRI of isolated hearts at 7 days after MI confirmed ^68^Ga‐FAPI‐04 accumulation within the infarcted myocardium. PET/CT scans showed the highest contrast in the infarcted myocardium at 7 days after MI, whereas ^68^Ga‐FAPI‐04 uptake in the infarcts was reduced to sham‐operation levels after simultaneous injection of non‐labeled FAPI. Furthermore, IHC staining revealed high expression of FAP within the infarct area. Overall, these results validated the specificity of the ^68^Ga‐FAPI signal to FAP and demonstrated its potential to reveal FAP expression in activated fibroblasts post‐MI.

FAP expression has been extensively studied in multiple diseases. FAP is widespread in the subpopulation of cancer‐associated fibroblasts (CAFs) in many epithelial cancers, which plays a pivotal role in tumor maintenance, growth, and migration [20]. In contrast to CAFs, normal fibroblasts have only minimal expression of FAP, leading to relatively low FAP expression levels in healthy tissues. FAP expression has also recently been demonstrated in non‐malignant pathological conditions, including wound repair [21], IgG4‐related disease [22], and renal fibrosis [23]. Notably, Tillmanns et al. demonstrated FAP expression in myocardial fibroblasts in MI rat hearts [9]. The expression levels of FAP, α‐SMA, vimentin, and periostin observed in the infarcts confirmed that the FAP^+^ cells in the infarcted myocardium were activated fibroblasts, consistent with previous reports [24, 25]. Furthermore, negligible expression of FAP and minimal uptake of ^68^Ga‐FAPI were observed in normal myocardium, suggesting the absence of fibroblast activation in healthy myocardial tissue. FAP expression could only identify activated fibroblasts but not resting fibroblasts. These results suggest that FAP may be a promising marker of myocardial fibrosis after MI through phenotypic changes in the fibroblasts.

Advances in fibroblast and fibrosis research have brought new approaches for the treatment of cardiac fibrosis. For example, Oatmen et al. noted that chemotherapeutic agents targeting the cardiac abnormal cardiac fibroblast phenotype might attenuate the development of myocardial fibrosis [26], while therapies targeting activated fibroblasts have been employed in liver fibrosis, lung fibrosis and other fibrotic diseases [27]. In these studies, timing appeared to be an important factor influencing the therapeutic effect. ^68^Ga‐FAPI‐04 is capable of assessing the status of activated fibroblasts through PET/CT and may serve as an auxiliary tool in the treatment of fibrotic diseases.

The specific expression pattern of FAP implies that it is a potential therapeutic target for diseases, and FAPI variants labeled with therapeutic radionuclides, such as ^177^Lu and ^225^Ac, have recently been assessed in different cancers [28, 29]. The conjunction of nuclide‐labeled FAPI with FAP‐expressing CAFs allows the targeted elimination of CAFs through therapeutic nuclides. Similarly, ^177^Lu‐FAPI‐04 may exert therapeutic effects by specifically targeting FAP expressed by activated fibroblasts following MI. In this study, cardiac function exhibited an increasing trend in rats in the 0.4 MBq/g group after ^177^Lu‐FAPI‐04 delivery. We then performed MI surgery on 6‐week‐old Wistar rats and administered ^177^Lu‐FAPI‐04 or 0.9% NaCl solution at 7 days after MI, to determine if treatment with the radionuclide could improve cardiac fibrosis following MI. Echocardiographic examination at 28 days after MI showed significantly improved left ventricular systolic function in the ^177^Lu‐FAPI group compared with vehicle controls. In addition, MRI and histological analysis confirmed a significant decrease in scar size in MI rats injected with ^177^Lu‐FAPI‐04. Based on the results of ^177^Lu‐FAPI therapy, we speculated that radiopharmaceutical inhibition of activated fibroblast might attenuate myocardial fibrosis and improve cardiac function in MI rats.

Pharmacological inhibition of FAP was also previously shown to mitigate myocardial fibrosis [30]. In addition, FAPI exerts its antifibrotic effect mainly by inhibiting excessive fibroblast activation. In our experiments, IF images at 7 days after ^177^Lu‐FAPI‐04 injection revealed significant differences in expression levels of periostin and vimentin between ^177^Lu‐FAPI and vehicle groups, although ^177^Lu‐FAPI did not significantly alter the expression of α‐SMA, indicating that ^177^Lu‐FAPI‐04 had influence on fibroblast activation. According to the study by Sun et al., continuous and high‐dose daily administration of FAPI after MI was required to achieve a therapeutic effect [30]. However, we administered only a single injection of ^177^Lu‐FAPI at 7 days after MI, and the total dose of FAPI was far below the level required for therapeutic effects. This further confirmed that the impact of ^177^Lu‐FAPI on fibroblast activation was achieved primarily through the radiation effects of ^177^Lu.

We further explored the mechanism by which ^177^Lu‐FAPI‐04 improved systolic function by detecting the expression of apoptotic proteins. MI rats showed significantly higher expression of apoptotic proteins compared with sham‐operated rats, suggesting the occurrence of apoptosis after MI. Apoptosis is a form of cell death controlled autonomously by intrinsic and extrinsic molecular pathways. The intrinsic pathway of apoptosis can be triggered by oxidative stress, radiation‐induced DNA damage, and other death stimuli [31]. Bax is an effector protein that triggers the intrinsic pathway of apoptosis through the formation of pores in the mitochondrial membrane. This pathway can be inhibited by pro‐survival proteins such as Bcl‐2, which serve as critical regulators of apoptosis [27]. In the absence of Bcl‐2, Bax becomes activated on the mitochondrial surface, leading to the formation of pores that facilitate mitochondrial outer membrane permeability and the subsequent release of apoptogenic molecules into the cell cytoplasm, such as second mitochondria‐derived activator of caspases and cytochrome c. Cytochrome c in the cytoplasm binds to apoptotic protease activating factor 1 to form the apoptosome, resulting in cell death via the activation of caspase‐3 and caspase‐7 [32]. Compared with vehicle group, ^177^Lu‐FAPI group showed higher Bax and Caspase‐3 levels and lower Bcl‐2 levels in α‐SMA^+^ fibroblasts, indicating the ability of ^177^Lu‐FAPI‐04 to induce fibroblast apoptosis. These results corroborated our hypothesis that ^177^Lu‐FAPI‐04 induces fibroblast apoptosis through ionizing radiation.

Biodistribution analysis of ^177^Lu‐FAPI‐04 showed increased activity in the liver and kidney 24 h after administration, and we therefore performed blood biochemical analysis. Rats in the ^177^Lu‐FAPI group showed no abnormal parameters compared with the vehicle group, indicating that ^177^Lu‐FAPI treatment did not produce significant hepatotoxicity or nephrotoxicity. Notably, however, this study only explored the short‐term toxicity of ^177^Lu‐FAPI‐04 at the 0.4 MBq level, and further studies are needed to assess the long‐term toxicity and the effect of higher doses of ^177^Lu‐FAPI‐04.

The treatment of myocardial fibrosis following MI remains a clinical challenge. Although reparative fibrosis is initially essential to avoid cardiac rupture, a prolonged fibrotic response in the non‐infarcted region may lead to cardiac dysfunction and HF [33]. ^68^Ga‐FAPI PET/CT provides a unique method for visualizing cardiac fibrotic progression, and may thus be able to identify a time window during which ^177^Lu‐FAPI‐04 may have a therapeutic effect in myocardial fibrosis. To date, anti‐fibrotic treatments targeting fibroblasts have been used to improve left ventricular function in preclinical studies [34, 35]. Elimination of FAP‐expressing activated fibroblasts by treatment with chimeric antigen receptor T cells has been shown to reduce fibrosis and improve cardiac function in mice [36], but depletion of stromal cells expressing FAP from bone marrow could induce cachexia and anemia [37]. In comparison, inhibition of FAP by ^177^Lu‐FAPI showed no significant side effects at the current dose and may thus be a safer approach for improving cardiac function and reducing myocardial fibrosis post‐MI.

This study had some limitations. First, we created an MI model using open‐chest surgery, and FAPI uptake in the surgical wound adjacent to the infarct area could potentially affect the diagnostic and therapeutic effects of ^68^Ga‐FAPI‐04 and ^177^Lu‐FAPI‐04. It is therefore necessary to establish a non‐invasive MI model for future research. Additionally, we conducted ^68^Ga‐ and ^177^Lu‐FAPI investigation after permanent ligation of the left anterior descending coronary artery (LAD) occlusion, and the results need to be confirmed in an ischemia‐reperfusion model. Finally, we did not incorporate various time points or employ multiple radionuclides for comparative analysis.

## 4 Conclusion

This study evaluated the ability of ^68^Ga‐FAPI‐04 PET/CT to monitor activated fibroblasts following MI and explored the feasibility and short‐term toxicity of ^177^Lu‐FAPI‐04 for the treatment of cardiac fibrosis after MI. ^177^Lu‐FAPI‐04 therapy can inhibit myocardial fibrosis by suppressing activation and inducing apoptosis of fibroblasts, and may thus contribute to the development of new strategies for the treatment of MI. Further studies are needed to investigate the feasibility of different doses and different therapeutic nuclides for the treatment of myocardial fibrosis post‐MI.

## 5 Methods

### 5.1 Establishment of MI Model

MI rats were established by permanent LAD. Briefly, male Wistar rats (6 weeks old, 200 g; Charles River, Beijing, China) were anesthetized by isoflurane inhalation (5%, 0.25 MPa, 1 L/min), followed by left thoracotomy and ligation of the LAD. Successful coronary occlusion was verified by electrocardiography within 2 h after surgery. Rats in the sham‐operated group underwent an equivalent surgical procedure, without ligation.

### 5.2 Radiolabeling

^68^Ga‐labeling of FAPI‐04 was accomplished using a ^68^Ge/^68^Ga generator (20 mCi; Cyclotron Co., Ltd, Obinisk, Russia). The generator was eluted with 0.1 M hydrochloric acid (5 mL), followed by the addition of [^68^Ga] GaCl_3_ solution (1000 µL, 160–200 MBq) into a mixture of FAPI‐04 (11.4 nmol, 88 µL, 0.1 mg/mL) and sodium acetate buffer (1000 µL, 0.1 M) at pH 4.5. The reaction system was heated to 95°C for 15 min followed by purification using a C18 extraction cartridge preconditioned with water (2.5 mL) and ethanol (1 mL). The cartridge was rinsed with ^68^Ga‐FAPI‐04 and saline (2 mL), and ^68^Ga‐FAPI‐04 was then eluted with ethanol (1 mL). Radio HPLC was carried out to determine the radiochemical purity of ^68^Ga‐FAPI‐04 (937.9 g/mol).

[^177^Lu] LuCl_3_ was procured from Chengdu Xinke Pharmaceutical Co., China. A solution of ^177^Lu in 0.05 M HCl (88 µL, 2375 MBq) was added into a mixture of FAPI‐04 (103.65 nmol, 0.1 mg/mL, 800 µL) and 0.1 M sodium acetate buffer (476 µL, pH 4.5). The reaction system was heated to 95°C for 20 min. The final product was diluted in 0.9% saline solution and filtered. The quality of ^177^Lu‐FAPI‐04 (1046.9 g/mol) was determined by HPLC.

### 5.3 ^68^Ga‐FAPI‐04 PET/CT

PET images were acquired using an Inveon PET/CT scanner (Siemens). Rats under 2% isoflurane anesthesia were injected with the radiotracer ^68^Ga‐FAPI‐04 via the tail vein. Static PET/CT images were acquired 50 min after tracer injection (3.7 MBq, 1, 3, 7, 14, and 28 days after coronary ligation) with an acquisition time of 10 min, and dynamic PET scans were started 7 days after ligation simultaneously with the bolus injection. The image was reconstructed using Siemens Inveon software with a three‐dimensional ordered subsets expectation maximum algorithm. Acquired data were reconstructed into 18 frames in the dynamic PET scan (5 min × 18 frames) and one frame in the static scan. In addition, circular regions of interest were outlined on axial PET images of the infarcted and remote myocardium to quantify ^68^Ga‐FAPI‐04 accumulation. ^68^Ga‐FAPI‐04 uptake was described as percent injected dose per gram of tissue (%ID/g). The infarct‐to‐remote ratio was interpreted as the ratio of the maximum ^68^Ga‐FAPI uptake in the infarcted area to the mean ^68^Ga‐FAPI uptake in a remote normal region.

### 5.4 Magnetic Resonance Imaging

MRI was carried out to evaluate the infarct area. Rats were sacrificed 7 days after surgery, 1 h after ^68^Ga‐FAPI‐04 injection, and their hearts were harvested and investigated using a 7 T MRI scanner (BioSpec 70/20 USR, Bruker, Germany). Data were acquired using T1 mapping (FLASH) with an acquisition time of 20 min. The same animals were scanned by ^68^Ga‐FAPI‐04 PET/CT before MRI. For ^177^Lu‐FAPI assessment, rats in different groups were sacrificed by euthanasia at 28 days after coronary ligation, and their hearts were removed and scanned by MRI, as above.

### 5.5 Echocardiography

Echocardiography was performed by a professional technician after electrocardiography validation. Rats were anesthetized with 2% isoflurane and placed on a heating pad, and continuous electrocardiography monitoring was carried out. Cardiac structure and function were assessed using an echocardiography device (Vinn06lab, Vinno, Suzhou, China), including LVEF, LVFS, LVEDV, and LVEDD.

### 5.6 Blocking Study

A blocking study was carried out to confirm the specificity of ^68^Ga‐FAPI‐04 accumulation in the infarcts. Briefly, rats in the blocked group were subjected to LAD ligation and co‐injected with ^68^Ga‐FAPI‐04 and excessive non‐labeled FAPI‐04 (200 nmol) to obstruct specific binding sites at 7 days after coronary ligation. In contrast, rats in the non‐blocked group were subjected to coronary ligation, and both the non‐blocked group and sham‐operated rats were injected with ^68^Ga‐FAPI‐04. The rats were then examined by PET/CT, and the hearts were removed for MRI and pathological staining.

### 5.7 ^177^Lu‐FAPI‐04 Treatment

^177^Lu‐FAPI‐04 was injected into male Wistar rats via the tail vein (7 weeks old, 230–270 g, 7 days after coronary ligation). In the concentration gradient experiment, MI rats injected with ^177^Lu‐FAPI‐04 were divided into three groups according to the injected dose: vehicle (0.9% NaCl solution), 0.2 MBq/g (51 ± 4 MBq), and 0.4 MBq/g (96 ± 4 MBq) groups. Echocardiography analysis was conducted before and 1 week after injection, and the rats were then sacrificed for pathological staining. An optimal dose of ^177^Lu‐FAPI was chosen for follow‐up experiments. In the following experiments, the new MI rats were injected with the effective dose of ^177^Lu‐FAPI (^177^Lu‐FAPI group) or normal saline (vehicle group) at 7 days after MI. Meanwhile, the sham‐operated rats were injected with normal saline (sham group).

### 5.8 Histological Examination

Rats were sacrificed at designated time‐points, and their hearts were resected and cut into serial 4‐µm short‐axis sections and used for HE, Masson, IHC, and IF staining. HE staining was conducted according to standard procedures. Masson staining was carried out using a Masson Trichrome Stain Kit, according to the manufacturer's directions (Solarbio, Beijing, China). Serial sections for IHC and IF were blocked with 10% goat serum (Proteintech, Wuhan, China) at room temperature for 20 min and incubated with primary antibody at 4°C overnight, followed by secondary antibody (Proteintech) at 37°C for 30 min. The slides were then incubated with DAPI (Beyotime, Shanghai, China) at room temperature for 10 min, sealed with anti‐fluorescence quenchers, and stored in the dark.

Antibodies used for these experiments were as follows: α‐SMA (Proteintech, 14395‐1‐AP), periostin (Proteintech, 19899‐1‐AP), vimentin (Proteintech, 10366‐1‐AP), cleaved caspase 3 (Proteintech, 68773‐1‐Ig), Bax (Proteintech, 60267‐1‐Ig), and Bcl‐2 (Proteintech, 26593‐1‐AP). The stained sections were imaged using a Nikon Eclipse 80i microscope and digital pathology slide scanner (DMS‐10‐Pro, d·metrix, Suzhou, China). FAP^+^, α‐SMA^+^, Vimentin^+^, and periostin^+^ cells were quantified using Image J (NIH).

### 5.9 Biodistribution and Toxicity

^177^Lu‐FAPI was injected into Wistar rats (0.4 MBq/g, 7 days after MI) to evaluate the whole‐body biodistribution. After euthanasia by deep inhalation anesthesia with isoflurane, the major organs, including heart, liver, spleen, kidney, lungs, and blood, were removed at 3 and 24 h post‐injection and activity was quantified using a 2470 Wizard Gamma Counter (Perkin Elmer). Activity counts were normalized by calibration using the ^177^Lu standard solution.

^177^Lu treatment‐induced toxicity was assessed by laboratory tests, including liver function tests (albumin and alanine aminotransferase) and renal function tests (blood urea nitrogen and creatinine), performed at 3, 24, and 168 h post‐radionuclide injection (7 days after coronary ligation).

### 5.10 Statistical Analysis

Statistical analysis was performed using SPSS 23.0 statistical software (IBM). Data were expressed as the mean ± standard deviation. An Independent‐samples Student's *t‐*test was used to compare two variables, and one‐way ANOVA was used to compare multiple variables. A *p*‐value < 0.05 was considered significant.

## Author Contributions

Y.Z. and X.S. performed the experiments and wrote the manuscript. B.X. and S.Z. performed the experiments and analyzed the data. X.Z. designed the study and revised the manuscript. All authors have read and approved the final manuscript.

## Ethics Statement

All animal studies were approved by the Institutional Animal Care and Ethics Committee of Nanjing Medical University (IACUC‐2409046).

## Conflicts of Interest

The authors declare no conflicts of interest.

## Data Availability

The corresponding author will supply the relevant data upon reasonable request.
